# Psychological Impact of the COVID-19 Pandemic on Dentists in Latin America’s Epicenter: São Paulo, Brazil

**DOI:** 10.3390/ijerph192215028

**Published:** 2022-11-15

**Authors:** Carlos Ariel Rodrigues de Araujo, Danielle Viana Ribeiro, Danielle Boina de Oliveira, Wander Barbieri, Gabriela Silva de Castilho, Manuel Jimenez, Tamara Kerber Tedesco, Maisa Camillo Jordão, Tatiane Fernandes Novaes, Danielle da Costa Palacio, Debora Heller

**Affiliations:** 1 Postgraduate Program in Dentistry, Universidade Cruzeiro do sul, São Paulo 08060-070, Brazil; 2 Hospital Israelita Albert Einstein, São Paulo 05652-900, Brazil; 3 Department of Physical Education and Health, Faculty of Education, International University of Lá Rioja, 26006 Logronõ, Spain; 4 Department of Periodontology, University of Texas Health Science Center at San Antonio, San Antonio, TX 78229, USA

**Keywords:** coronavirus, SARS-CoV-2, COVID-19, dentistry, mental health, stress, anxiety

## Abstract

The present study aimed to assess the prevalence and associated factors of stress and anxiety symptoms among dentists during the COVID-19 pandemic in the state of São Paulo, Brazil. A structured questionnaire was sent electronically to 93,280 dentists with active registration in the Dental Council of São Paulo, Brazil, enquiring about information regarding the first-wave peak period in Brazil. Descriptive analyses of background characteristics, perceptions of preparedness, and psychological impact were calculated. Multiple logistic regression analysis was performed, and independent variables that showed *p* < 0.20 were used in the adjusted logistic regression model to compare the psychological impact on dental professionals. Among the 2113 respondents, female participants had 63% lower chance of reporting anxiety than males. Older dentists had a lower likelihood of reporting anxiety compared to 21–30-year-old dentists (*p* ≤ 0.05). Dentists working in the public health service were 1.78 times more likely to report anxiety than those who worked in private practice. Finally, dentists in the COVID-19 high-risk group and those with a family or team member with a positive COVID-19 diagnosis were more likely to have anxiety. This study can help dental and other healthcare professionals to better understand the consequences of COVID-19 in terms of mental health.

## 1. Introduction

The fear of becoming infected with COVID-19 in the dental office and taking the disease home, in addition to the socioeconomic impact caused by the pandemic [1], exposed dental professionals to mental disorders. After more than two years of SARS-CoV-2 circulation around the world, dental practice is still seen as high risk and stigmatized by the global health crisis.

Dentists are at higher risk of contagion during their routine procedures [2,3], with the risk of potentially transmitting it to their peers, families, and patients. SARS-CoV-2 transmission during dental procedures can happen through exposure to saliva and blood, the inhalation of aerosol/droplets from infected individuals, or direct contact with mucous membranes, oral fluids, and contaminated surfaces and instruments [4,5,6]. In this context, in early 2020, regulatory legislations regarding restrictions on dental care were issued by different countries, including the United States and Brazil [7,8], forcing the public and private dental health systems to make adjustments that many times included dental practice closures for different periods of time.

This contagious virus has not only raised concerns over general public health, but has also caused several psychological and mental disorders in individuals and different communities worldwide [9]. An expressive mental health burden has been imposed on society as a whole, and particularly on healthcare workers [9,10]. Studies conducted recently pointed out that healthcare professionals have a higher risk of developing mental health problems and an increased prevalence of depression, anxiety, and insomnia [11]. In the UK, the British Dental Association showed a 575% increase in the percentage of dentists seeking mental health counseling [12]. In addition, when anxiety and fear regarding COVID-19 were evaluated among dentists from 30 different countries (Saudi Arabia, Pakistan, India, the United Arab Emirates, China, Italy, the United Kingdom, Australia, Malaysia, the United States of America, Ireland, Israel, New Zealand, South Africa, Turkey, Germany, Kuwait, Canada, Hungary, France, Poland, Bulgaria, the Republic of the Congo, Mexico, Romania, Egypt, Switzerland, Demark, and Bahrain), it was observed that more than 72% of professionals felt an increase in the fear of close contact with patients in clinical practice, which led to increased stress and 66% of dentists reporting a desire to leave the profession [13]. Moreover, additional studies have reported an increase in the number of professionals reporting feeling fearful and seeking counseling [14,15,16,17,18].

Although poor mental health and wellbeing are often noted during a pandemic, adequate resources to limit and/or prevent negative psychological effects are lacking [19]. Attention to mental health is commonly superseded by urgent health needs, such as immediately caring for people with the disease, as well as testing, monitoring, and preventing it. However, the understanding of how healthcare professionals’ mental health is impacted during a pandemic is critical to avoid burnout, and further help preserve the long-term stability and effectiveness of healthcare systems [20].

Previous studies confirmed that the COVID-19 pandemic had a negative psychological impact on dentists worldwide. However, different levels of psychological distress can be observed in different countries due to social, cultural, and environmental issues [11,12,16,17,21,22,23,24,25,26,27,28,29,30,31].

There are 369,716 registered dentists in Brazil, the country with the highest number of dentists in the world, and over 93 thousand of these dental professionals are registered in the state of São Paulo [32]. It is not known how dentists in São Paulo, Brazil, experienced the pandemic on a psychological level. Thus, this study aimed to assess the prevalence and associated factors of stress and anxiety symptoms among dentists during the COVID-19 pandemic in the state of São Paulo, Brazil.

## 2. Methods

### 2.1. Ethical Aspects and Study Design

This study was approved by the Universidade Cruzeiro do Sul institutional ethics committee (protocol #31720720.9.0000.8084), and was conducted according to the Helsinki declaration. The consent form was made available through an alternative in the online form before the questionnaire.

A questionnaire-based cross-sectional study was conducted and reported according to the SURGE guideline (the Survey Reporting Guideline) [33] and STROBE Statement [34] (Table S1).

### 2.2. Setting and Participants

A semi-structured questionnaire was sent to all dentists with active registration in the Regional Dental Council of São Paulo (CROSP), Brazil, who had an email address cataloged in their database. An Instagram post and WhatsApp messages were also sent to enhance dentists’ participation and encourage them to check their inboxes.

Only dentists who agreed to participate were included in the study.

The questionnaire was hosted online (Google Forms) for 8 days (from 25 June 2020, up to 02 July 2020).

### 2.3. Data Collection Instrument 

For mental health, a validated Depression Anxiety and Stress Scale (DASS-21—short-form adaptation and validation for Brazilian adolescents) was administered [35]. The other questions were constructed based on previously published questionnaires. The questionnaire consisted of 16 questions, divided into three main groups: Dimension 1—Sociodemographic characteristics of the interviewed population and contact with the SARS-CoV-2 virus (eight questions); Dimension 2—Education characteristics of the interviewed population (four questions); and Dimension 3—Psychological impact (stress and anxiety) and work characteristics (four questions). The questionnaire is presented in Tables S2 and S3. To certify the good reliability and validity of the questionnaire and its dimensions, we conducted a pre-test in a convenience sample of 10 dentists. The pre-testers were asked to answer the questionnaire, give their feedback on its clarity, and record the time spent completing all questions. After receiving their feedback, only small wording changes as well as adjustments to the questions’ sequence were made in order to improve the clarity of the questionnaire. The pre-testers were not eligible to participate in this study, since they were dentists from the public and private sectors from other states in Brazil.

### 2.4. Statistical Analysis

Descriptive analysis was used to describe the sociodemographic and work characteristics of the participants (*n*/%). Multiple logistic regression analysis was used to evaluate the association between the dependent variables—anxiety and stress—with the independent variables (sex, age range, IBGE race classification, higher education level, time since graduation, place of residence, main workplace, income reduction, COVID-19 high-risk group (as defined by the World Health Organization [WHO]) [36], positive COVID-19 diagnosis, family or team member with a positive COVID-19 diagnosis, appointment with a psychologist or psychiatrist in the last 30 days). It was considered a well-used strategy for sample size, which aims to ensure at least 20 events per candidate predictor (variable), i.e., those that are considered potentially predictive of the outcome [37,38].

Initially, an unadjusted logistic regression analysis was performed for each of the independent variables, with those that showed *p* < 0.20 being included in the adjusted logistic regression model. Only the variables that showed *p* < 0.05 remained in the final model. The data were analyzed in the Jamovi Project (Jamovi version 1.6, Sydney, Australia, 2021). The statistical analysis evaluated the null hypothesis that the independent variables—sex, age range, IBGE race classification, higher education level, time since graduation, place of residence, main workplace, income reduction, COVID-19 high-risk group, positive COVID-19 diagnosis, family or team member with a positive COVID-19 diagnosis, appointment with a psychologist or psychiatrist in the last 30 days—are not related with the self-perception of stress or anxiety symptoms.

## 3. Results

The structured questionnaire was sent electronically to all 93,280 dentists actively registered with the Dental Council of São Paulo, Brazil (CROSP). Of those, 2348 accessed the online Google Forms, and 2113 accepted the invitation to participate after reading the informed consent form. The non-response rate was 10% (*n* = 235). Table 1 displays the sociodemographic and work characteristics of the participants. Most of the respondents were male (74.1%), living in the countryside (56.7%), with ages ranging from 41 to 50 years old (32.4%), and of the white race (86.9%). Regarding their higher educational level, 70.9% reported having a postgraduate degree, having more than 20 years of clinical experience (43.2%), and a current main workplace of a private practice (73.9%).

Table 2 shows the results from the multiple logistic regression analysis for the anxiety variable. The IBGE race classification, higher education level, time since graduation, city, and positive COVID-19 diagnosis were not associated with anxiety.

Female participants had 63% (OR = 0.377; 95% CI 0.300–0.423) lower chance of reporting anxiety than males. Moreover, the age range also influenced anxiety, with older dentists demonstrating a lower likelihood of reporting anxiety compared to 21–30-year-old dentists (*p* ≤ 0.05).

Regarding their work characteristics, dentists who worked predominantly in public dental service were 1.78 times more likely to report anxiety than those working in private practice (OR = 1.780; 95% CI 1.169–2.708). Similarly, when there was an income reduction of more than 50%, the participants were 1.46 times more likely to report anxiety than when there was less than 10% or no income reduction (OR = 1.476; 95% CI 1.087–2.004).

Furthermore, dentists in the COVID-19 risk group (OR = 1.449; 95% CI 1.150–1.826) and those with a family or team member with a positive COVID-19 diagnosis (OR = 1.449; 95% CI 1.150–1.826) were more likely to have anxiety. In addition, the participants who reported having an appointment with a psychologist or psychiatrist in the last 30 days were more likely to report anxiety than those who did not have an appointment in this period (OR = 158.398; 95% CI 22.113–1134.592).

Table 3 shows the results from the multiple logistic regression analysis for the stress variable. The IBGE race classification, higher education level, city, main workplace, positive COVID-19 diagnosis, and to have reported having an appointment with a psychologist or psychiatrist in the last 30 days were not associated with stress.

Female participants had 66% (OR = 0.327; 95% CI 0.252–0.423) lower chance to report stress than males. Moreover, the age range also influenced the presence of stress, being that older dentists were less likely to report stress compared to 21–30-year-old dentists (*p* ≤ 0.05).

Income was also influenced by the presence of stress, whereas dentists with an income reduction of more than 50% were 1.57 more likely to report stress (OR = 1.570; 95% CI 1.116–2.206). Similarly, dentists in the COVID-19 risk group (OR = 1.428; 95% CI 1.010–2.019) and those with a family or team member with a positive COVID-19 diagnosis (OR = 1.389; 95% CI 1.042–1.851) were more likely to report stress.

## 4. Discussion

During the COVID-19 pandemic, dentists worldwide experienced a period of uncertainty and insecurities, putting them at increased risk of mental health problems [6,12,14,15,16,18,27,29,30,39,40,41]. The present study demonstrated the levels of stress and anxiety in dentists working in the state of São Paulo during the SARS-CoV-2 viral outbreak. To the best of our knowledge, this is the first study to evaluate the emotional impact and psychological symptoms among Brazilian dentists. High rates of mental disorders were observed among health professionals in many countries, including China, the USA, Hungary, Turkey, India, and Italy [21,22,23,26,41,42,43,44,45,46,47]. However, few studies have analyzed the psychological impact on dentists, commonly restricted to a specific dental specialty [6,16,48] or focused on dental care [49].

Knowing that SARS-CoV-2 is transmitted predominantly through aerosol and salivary droplets [4] and that dental interventions are performed close to the oropharyngeal region, dentists are exposed to a higher risk of infection [50], becoming a potential carrier, but also a transmitter of the disease, if the current biosafety recommendations are not followed. These facts, added to uncertainty or lack of knowledge about the pathogenesis of COVID-19 and its social and economic impacts [1], were factors that justify its negative psychological effect on dental professionals [51].

In this study, a questionnaire with closed questions was used to assess the psychological impact on dentists working in the state of São Paulo, Brazil. While different instruments have been applied to assess the psychological impact imposed by COVID-19 worldwide, in the present study, the choice of the “Depression Anxiety and Stress Scale” is justified because it involves a theoretical model that discriminates well the symptoms of stress and anxiety, which are not always differentiated by other scales. Additionally, the applied version is the only one validated for Brazilian Portuguese, being a compact version, which is comprehensive and easy to apply [35,52].

Female participants were less likely to experience anxiety and stress than male participants. In contrast, other studies revealed no differences between women and men, as well as in UK frontline health workers, where no differences were identified between the sexes [23,29]. Several studies revealed that women had significantly higher levels of self-reported anxiety [4,6], and also confirmed that women were at greater risk of depressive symptoms than men. Importantly, our findings should be treated with caution, as most respondents were male (74.1%).

As for the sociodemographic characteristics evaluated, it is possible to state that the demographic characteristics of the sample studied are representative of the entire population of dentists in São Paulo [53,54,55]. Older participants were less likely to report experiencing anxiety and stress compared to dentists aged between 21 and 30 years old. This corroborates with studies carried out with dentists in Asia, Europe, and other countries in Latin America [29,56] where younger dentists reported a higher level of stress and anxiety. This finding could be explained by the guidance to suspend elective dental care by national and international health entities (Ministry of Health, ANVISA, and the WHO) for long periods of 2020 [36,57].

As for the workplace, dentists in the public network were 1.78 times more likely to have anxiety than those in the private network. These data, when compared to other countries, show controversial results [41]. This can be explained by the characteristic of the organization of public services in the state of São Paulo, where dentists acted on the front line in facing the pandemic, working outside of their specialty, acting on COVID-19 triage, telemedicine, and, later on, vaccination.

As for studies that showed a higher level of stress among professionals in the private sector, this may be related to the financial impact generated by the suspension of clinical care [41], which was also observed in the present study.

One aspect that can be considered a limitation of the study is the regional issue, as the questionnaire was only distributed to dentists in the State of São Paulo, obtaining 2113 responses. Even though all registered dentists in the state of São Paulo were invited to participate, a lower response rate was achieved. The participants who promptly responded to the present questionnaire could be those who felt most affected/impacted by the pandemic. In fact, volunteer bias is an inherent limitation of this study design. However, our results are in agreement with studies conducted in Europe and the United States that confirmed the negative mental impact of the COVID-19 pandemic on other populations. Currently, global cooperation towards pandemic preparedness is lagging, and sharing data and scientific evidence gathered from different settings and in different countries around the world is an important step that needs to be taken to change course [58]. Future studies should include different populations and longitudinal evaluations.

The present study highlights the negative impact of the pandemic on the mental health of dentists. Even two years after the World Health Organization (WHO) characterized the global spread of COVID-19 as a pandemic, governments and health professionals worldwide should take these lessons on board as we shape public health messages for our post-COVID-19 world and be better prepared when the next pandemic arrives.

## 5. Conclusions

The COVID-19 pandemic resulted in a negative psychological impact on dentists in the state of São Paulo. With the COVID-19 pandemic and all of the post-traumatic effects that followed the disease, we showcase the importance of establishing mental health support systems for dental professionals to better respond to the impact of this and future pandemics.

## Figures and Tables

**Table 1:** Sociodemographic and work characteristics of the participants (*n* = 2106).

Variables		*n*	%
Sex	Male	1561	74.1
	Female	544	25.8
	Other	1	0.1
Age range	21–30	397	18.9
	31–40	516	24.5
	41–50	683	32.4
	51–60	378	17.9
	>60	132	6.3
IBGE race classification	White	1830	86.9
	Brown/Black	133	6.3
	East Asian	126	6.0
	I prefer not to answer	17	0.8
Higher education level	Dentistry degree	613	29.1
	Postgraduate degree (certificate/MSc/PhD/Postdoc)	1493	70.9
Time since graduation (clinical experience in years)	<4 years/not working in clinical practice	342	16.2
	5–10 years	297	14.1
	11–20 years	557	26.4
	>20 years	910	43.2
Lives in	São Paulo	911	43.3
	Countryside	1195	56.7
Main workplace	Private practice	1556	73.9
	Hospital	100	4.7
	University/Research laboratory	224	10.6
	Public dental service	226	10.7

**Table 2:** Descriptive and logistic regression analysis (odds ratio; 95% confidence interval) of anxiety and independent variables.

Variables		Anxiety *n* (%)	Unadjusted OR (95% CI)	*p*-Value	Adjusted OR (95% CI)	*p*-Value
Sex	Male	1164 (74.6%)	Ref.	<0.001	Ref.	
	Female	263 (48.3%)	0.319 (0.261–0.391)		0.377 (0.300–0.423)	<0.001
	Other	0 (0%)	1.19 × 10^−6^ (4.50 × 10^−283^–3.15 × 10^270^)	0.966	3.96 × 10^−6^ (1.50 × 10^−282^–1.05 × 10^271^)	0.969
Age range	21–30	328 (82.6%)	Ref.	<0.001	Ref.	
	31–40	374 (72.5%)	0.554 (0.400–0.766)		0.442 (0.281–0.695)	<0.001
	41–50	443 (64.9%)	0.388 (0.286–0.526)	<0.001	0.410 (0.242–0.693)	<0.001
	51–60	229 (60.6%)	0.323 (0.232–0.450)	<0.001	0.412 (0.227–0.747)	0.003
	>60	53 (40.2%)	0.141 (0.091–0.218)	<0.001	0.175 (0.086–0.355)	<0.001
IBGE race classification	White	1224 (68.0%)	Ref.	0.058	Ref.	
	Brown/Black	101 (75.9%)	1.487 (0.987–2.239)		1.087 (0.686–1.722)	0.723
	East Asian	76 (60.3%)	0.716 (0.494–1.037)	0.077	0.681 (0.448–1.032)	0.070
	I prefer not to answer	6 (35.3%)	0.257 (0.094–0.698)	0.008	0.395 (0.133–1.172)	0.094
Higher education level	Dentistry degree	438 (71.5%)	Ref.		Ref.	
	Postgraduate degree (certificate/ MSc/PhD/ postdoc)	989 (66.2%)	0.784 (0.638–0.963)	0.020	0.859 (0.663–1.112)	0.248
Time since graduation (clinical experience in years)	< 4 years/not working in clinical practice	266 (77.8%)	Ref.	0.918	Ref.	
	5–10 years	232 (78.1%)	1.020 (0.701–1.484)		1.368 (0.862–2.171)	0.183
	11–20 years	401 (72.0%)	0.734 (0.536–1.006)	0.055	1.391 (0.846–2.288)	0.193
	> 20 years	528 (58.0%)	0.395 (0.296–0.526)	<0.001	1.019 (0.582–1.782)	0.947
Lives in	São Paulo	628 (68.9%)	Ref.			
	Countryside	799 (66.9%)	0.909 (0.756–1.09)	0.313	-	-
Main workplace	Private practice	1044 (67.1%)	Ref.		Ref.	
	Hospital	67 (67%)	0.996 (0.648–1.53)	0.984	1.369 (0.844–2.220)	0.203
	University/Research laboratory	145 (64.7%)	0.900 (0.671–1.21)	0.483	1.108 (0.785–1.564)	0.558
	Public dental service	171 (75.7%)	1.525 (1.105–2.10)	0.010	1.780 (1.169–2.708)	0.007
Income reduction	No income reduction/ less than 10%	390 (69.6%)	Ref.		Ref.	
	Income reduction between 10% and 50%	518 (64.9%)	0.806 (0.640–1.02)	0.068	1.230 (0.914–1.653)	0.171
	More than 50% income reduction	519 (69.4%)	0.988 (0.779–1.25)	0.920	1.476 (1.087–2.004)	0.012
COVID-19 high-risk group	No	1060 (68.1%)	Ref.		Ref.	
	Yes	343 (66.0%)	0.909 (0.736–1.12)	0.372	1.584 (1.202–2.086)	0.001
	I do not know	24 (82.8%)	2.251 (0.854–5.93)	0.101	2.891 (1.038–8.050)	0.042
Positive COVID-19 diagnosis	No	1379 (67.4%)	Ref.		Ref.	
	Yes	48 (78.7%)	1.78 (0.960–3.31)	0.067	1.404 (0.723–2.727)	0.316
Family or team member with a positive COVID-19 diagnosis	No	805 (62.9%)	Ref.		Ref.	
	Yes	533 (74.0%)	1.68 (1.37–2.06)	<0.001	1.449 (1.150–1.826)	0.002
	I do not know	89 (84.0%)	3.09 (1.82–5.25)	<0.001	2.651 (1.498–4.693)	<0.001
Appointment with a psychologist or psychiatrist in the last 30 days	No	1136 (62.6%)	Ref.		Ref.	
	Yes	291 (99.7%)	173.68 (24.33–1239.92)	<0.001	158.398 (22.113–1134.592)	<0.001

**Table 3:** Descriptive and logistic regression analysis (odds ratio; 95% confidence interval) of stress and independent variables.

Variables		Stress *n* (%)	Unadjusted OR (95% CI)	*p*-Value	Adjusted OR (95% CI)	*p*-Value
Sex	Male	1401 (89.8%)	Ref.	<0.001	Ref.	
	Female	388 (71.3%)	0.284 (0.222–0.364)		0.327 (0.252–0.423)	<0.001
	Other	1 (100%)	32,738.115 (1.24 × 10^−272^–866 × 10^280^)	0.974	99430 (3.76 × 10^−272^–263 × 10^281^)	0.972
Age range	21–30	363 (91.4%)	Ref.	0.002	Ref.	
	31–40	453 (87.8%)	0.527 (0.350–0.794)		0.508 (0.287–0.898)	0.020
	41–50	580 (84.9%)	0.164 (0.099–0.270)	<0.001	0.477 (0.247–0.920)	0.027
	51–60	310 (82.0%)	0.673 (0.434–1.045)	0.078	0.466 (0.224–0.970)	0.041
	>60	84 (63.6%)	0.427 (0.275–0.662)	<0.001	0.157 (0.068–0.361)	<0.001
IBGE race classification	White	1550 (84.7%)	Ref.	0.087	Ref.	
	Brown/Black	120 (90.2%)	1.667 (0.928–2.997)		1.432 (0.770–2.661)	0.257
	East Asian	109 (86.5%)	1.158 (0.684–1.961)	0.585	1.209 (0.697–2.097)	0.499
	I prefer not to answer	11 (64.7%)	0.331 (0.121–0.903)	0.031	0.349 (0.119–1.023)	0.055
Higher education level	Dentistry degree	533 (86.9%)	Ref.		Ref.	
	Postgraduate degree (certificate/ MSc/PhD/ postdoc)	1257 (84.2%)	0.799 (0.608–1.05)	0.108	0.892 (0.646–1.229)	0.484
Time since graduation (clinical experience in years)	<4 years/not working in clinical practice	302 (88.3%)	Ref.	0.520	Ref.	
	5–10 years	267 (89.9%)	1.179 (0.714–1.946)		1.580 (0.879–2.839)	0.126
	11–20 years	492 (88.3%)	1.003 (0.659–1.524)	0.991	1.937 (1.058–3.543)	0.032
	> 20 years	729 (80.1%)	0.533 (0.369–0.771)	<0.001	1.399 (0.714–2.736)	0.327
Lives in	São Paulo	774 (85.0%)	Ref.			
	Countryside	1016 (85.0%)	1.00 (0.789–1.28)	0.970	-	-
Main workplace	Private practice	1323 (85.0%)	Ref.			
	Hospital	89 (89%)	1.425 (0.750–2.71)	0.279		
	University/Research laboratory	186 (83%)	0.863 (0.592–1.26)	0.439	-	-
	Public dental service	192 (85%)	0.995 (0.673–1.47)	0.978		
Income reduction	No income reduction/ less than 10%	474 (84.6%)	Ref.		Ref.	
	Income reduction between 10% and 50%	620 (82.7%)	0.868 (0.647–1.16)	0.344	1.023 (0.747–1.399)	0.889
	More than 50% income reduction	656 (87.7%)	1.294 (0.942–1.78)	0.111	1.570 (1.116–2.206)	0.009
COVID-19 high-risk group	No	1333 (85.6%)	Ref.		Ref.	
	Yes	430 (82.7%)	0.803 (0.614–1.05)	0.108	1.428 (1.010–2.019)	0.044
	I do not know	27 (93.1%)	2.269 (0.536–9.61)	0.266	2.229 (0.508–9.774)	0.288
Positive COVID-19 diagnosis	No	1736 (84.9%)	Ref.			
	Yes	54 (88.5%)	1.37 (0.619–3.05)	0.435	-	-
Family or team member with a positiveCOVID-19 diagnosis	No	1058 (82.7%)	Ref.		Ref.	
	Yes	634 (88.1%)	1.55 (1.18–2.02)	0.001	1.389 (1.042–1.851)	0.025
	I do not know	98 (92.5%)	2.57 (1.23–5.36)	0.012	2.070 (0.965–4.439)	0.062
Appointment with a psychologist or psychiatrist in the last 30 days	No	1498 (82.6%)	Ref.			
	Yes	292 (100%)	2.44 × 10^7^ (3.00× 10^−318^-Inf.)	0.964	-	-

## Data Availability

All relevant data are within the manuscript and its Supporting Information files.

## Supplementary Materials

The following supporting information can be downloaded at: https://www.mdpi.com/article/10.3390/ijerph192215028/s1, Table S1: STROBE Statement—Checklist of items that should be included in reports of cross-sectional studies; Table S2: Questionnaire (original language: Brazilian Portuguese); Table S3: Translated Questionnaire (English).
