# Supplementary material for: Psychological Impact of the COVID-19 Pandemic on Dentists in Latin America’s Epicenter: São Paulo, Brazil
Source: Int J Environ Res Public Health. 2022 Nov 15;19(22):15028. doi: 10.3390/ijerph192215028 (PMC9691075; doi:10.3390/ijerph192215028)
Supplement: Supplementary file 1 [file ijerph-19-15028-s001.zip › ijerph-1813223-supplementary.pdf]

Table S1. STROBE Statement—Checklist of items that should be included in reports of ***cross-sectional studies.***

	Item No	Recommendation	Page
**Title and abstract**	1	(*a*) Indicate the study's design with a commonly used term in the title or the abstract	2
		(*b*) Provide in the abstract an informative and balanced summary of what was done and what was found	2
**Introduction**			
Background/rationale	2	Explain the scientific background and rationale for the investigation being reported	3/4
Objectives	3	State specific objectives, including any prespecified hypotheses	4
**Methods**			
Study design	4	Present key elements of study design early in the paper	4
Setting	5	Describe the setting, locations, and relevant dates, including periods of recruitment, exposure, follow-up, and data collection	4
Participants	6	(*a*) Give the eligibility criteria, and the sources and methods of selection of participants	4
Variables	7	Clearly define all outcomes, exposures, predictors, potential confounders, and effect modifiers. Give diagnostic criteria, if applicable	5
Data sources/ measurement	8*	For each variable of interest, give sources of data and details of methods of assessment (measurement). Describe comparability of assessment methods if there is more than one group	5
Bias	9	Describe any efforts to address potential sources of bias	5
Study size	10	Explain how the study size was arrived at	4
Quantitative variables	11	Explain how quantitative variables were handled in the analyses. If applicable, describe which groupings were chosen and why	5
Statistical methods	12	(*a*) Describe all statistical methods, including those used to control for confounding	5
		(*b*) Describe any methods used to examine subgroups and interactions	NA
		(*c*) Explain how missing data were addressed	NA
		(*d*) If applicable, describe analytical methods taking account of sampling strategy	NA
		(*e*) Describe any sensitivity analyses	NA
**Results**			
Participants	13*	(a) Report numbers of individuals at each stage of study—eg numbers potentially eligible, examined for eligibility, confirmed eligible, included in the study, completing follow-up, and analysed	5
		(b) Give reasons for non-participation at each stage	5
		(c) Consider use of a flow diagram	NA
Descriptive data	14*	(a) Give characteristics of study participants (eg demographic, clinical, social) and information on exposures and potential confounders	5/Table 1
		(b) Indicate number of participants with missing data for each variable of interest	NA
Outcome data	15*	Report numbers of outcome events or summary measures	7
Main results	16	(*a*) Give unadjusted estimates and, if applicable, confounder-adjusted	Table

		estimates and their precision (eg, 95% confidence interval). Make clear which confounders were adjusted for and why they were included	2/3
		(*b*) Report category boundaries when continuous variables were categorized	NA
		(*c*) If relevant, consider translating estimates of relative risk into absolute risk for a meaningful time period	NA
Other analyses	17	Report other analyses done—eg analyses of subgroups and interactions, and sensitivity analyses	NA
**Discussion**			
Key results	18	Summarise key results with reference to study objectives	14
Limitations	19	Discuss limitations of the study, taking into account sources of potential bias or imprecision. Discuss both direction and magnitude of any potential bias	15
Interpretation	20	Give a cautious overall interpretation of results considering objectives, limitations, multiplicity of analyses, results from similar studies, and other relevant evidence	15
Generalisability	21	Discuss the generalisability (external validity) of the study results	15
**Other information**			
Funding	22	Give the source of funding and the role of the funders for the present study and, if applicable, for the original study on which the present article is based	15

*Give information separately for exposed and unexposed groups.

Note: An Explanation and Elaboration article discusses each checklist item and gives methodological background and published examples of transparent reporting. The STROBE checklist is best used in conjunction with this article (freely available on the Web sites of PLoS Medicine at http://www.plosmedicine.org/, Annals of Internal Medicine at http://www.annals.org/, and Epidemiology at http://www.epidem.com/). Information on the STROBE Initiative is available at www.strobe-statement.org.

Table S2. Questionnaire (original language: Brazilian Portuguese)

DIMENSÃO 1 – CARACTERÍSTICAS GERAIS DA POPULAÇÃO ENTREVISTADA

Email: (Opcional).

1. Qual cidade você mora?

2. Qual cidade você trabalha?

3. Como você se indentifica?

( ) Homem
( ) Mulher
( ) Outro

4. De acordo com as categorías do censo do IBGE para raça ou cor, você se declara:

( ) Indigena
( ) Amarelo (a)
( ) Preto (a)
( ) Pardo (a)
( ) Branco(a)
( ) Prefiro não responder

5. Faixa etária

( ) 21 - 30 anos
( ) 31 - 40 anos
( ) 41 - 50 anos
( ) 51 - 60 anos
( ) Acima de 60 anos

6. Você desenvolveu algum sinal e/ou sintoma do COVID-19?

( ) Tive sintomas
( ) Não tive sintomas
( ) Não sei se tive sintomas

7. Você foi diagnosticado com o vírus (SARS-CoV-2)?

( ) Sim, fiz o teste e o diagnóstico foi positivo
( ) Não, fiz o teste e o diagnóstico foi negativo
( ) Tive sintomas mas não consegui fazer o teste

8. Alguém da sua família ou equipe de trabalho desenvolveu algum sintoma ou foi diagnosticado com COVID-19?

( ) Sim
( ) Não
( ) Não sabe

DIMENSÃO 2 – CARACTERÍSTICAS EDUCACIONAIS DA POPULAÇÃO ENTREVISTADA

1. Área de atuação: (Você pode escolher mais de uma opção como resposta)

( ) Dentística Restauradora
( ) Odontologia Legal
( ) Pediatria
( ) Ortodontia e Ortopedia Facial
( ) Patologia Bucal
( ) Prótese Buco Maxilo Facial
( ) Prótese Dentária
( ) Saúde Coletiva e da Família
( ) Radiologia Odontológica e Imaginologia
( ) Disfunção Temporo-Mandibular
( ) Odontologia do Trabalho
( ) Odontologia para Pacientes com Necessidades Especiais
( ) Ortopedia Funcional dos Maxilares
( ) Gestão
( ) Odontologia Hospitalar
( ) Ensino

2. Grau de formação concluído:

( ) Clínico Geral
( ) Especialista
( ) Mestrado
( ) Doutorado
( ) Pós-doutorado

3. Qual seu tempo de experiência no trabalho ou na especialidade q você exerce?

( ) Até 4 anos
( ) 5 a 10 anos
( ) 11 a 20 anos
( ) 21 ou mais
( ) Não atuo clinicamente

4. Onde você trabalha? (Você pode escolher mais de uma opção como resposta)

( ) Hospital Publico
(...) Hospital privado
(...) UBS/AMA
(...) Clínica particular
(...) Policlínica
(...) Laboratório de Pesquisa
(...) Clínica Escola/ Faculdade
(...) Outro

5. Conhece os protocolos de atendimento e biossegurança relacionado ao COVID-19?

( ) Sim, todos
( ) Alguns
( ) Apenas dentro da minha especialidade
( ) Quase nada
( ) Nenhum

6. Você recebeu algum treinamento para o uso, manutenção e descarte de EPIs? Se sim, onde foi esse treinamento? Mais de uma opção poderá ser marcada. Se você marcou "não" na pergunta acima, marque "não se aplica".

( ) Sim
( ) Não
( ) Graduação
( ) Pós-graduação
( ) Na instituição/local em que trabalha
( ) Em outra instituição que já trabalhou
( ) Por conta própria
( ) Vi um vídeo de treinamento
( ) Sigo os protocolos de um Ebook
( ) Não se aplica

7. Você consulta os sites dos conselhos de classe (CRO, CFO, entre outros) para busca de informações, protocolos clínicos e de biossegurança relacionados ao COVID – 19 ?

( ) Sim
( ) Não

8. Onde você costuma buscar informações sobre Biossegurança e Descarte de Resíduos de Saúde?

( ) Livros
( ) Periódicos
( ) Protocolos dos Conselhos de Classe
( ) Internet
( ) Outros

DIMENSÃO 3 – CARACTERÍSTICAS CLÍNICAS / DE TRABALHO E VARIÁVEIS ECONÔMICAS

1. Se sente apto a atender seus pacientes com estas informações?

( ) Sim, totalmente
( ) Sim, mas com dúvidas
( ) Não

2. Qual alternativa descreve o que você sente quanto ao seu trabalho; mais de uma opção pode ser marcada como resposta:

( ) Preciso voltar ao trabalho para poder pagar minhas contas
( ) Preciso voltar ao trabalho por que eu amo minha profissão e não consigo ficar sem trabalhar, em casa
( ) Só vou voltar ao trabalho se meu patrão exigir ou cortar meu salário
( ) Tenho medo de pegar a doença atendendo meus pacientes
( ) Não tenho medo de pegar a doença atendendo meus pacientes
( ) Não parei de trabalhar durante a Pandemia

3. Na sua rotina de atendimento, com relação ao uso de EPIs, assinale o que é verdadeiro:

RESPOSTAS EM BOLINHAS
Não uso
Já utilizava e vou continuar a utilizar
Passei a utilizar após a pandemia
Tenho utilizado por falta de outra opção no mercado

Máscara cirúrgica, com tripla em TNT
Máscara N95
Máscara de tecido
Gorro descartável
Gorro de tecido
Óculos de proteção
Luvas de procedimento (descartáveis)
Avental de tecido
Avental descartável
Máscara facial acrílica/ FACE SHIELD
Pro pé
Luva Cirúrgica
Outros__________________________

4. O que você tem feito, ou considera importante fazer no ambiente de atendimento odontológico, como medida de prevenção na transmissão do coronavírus (Você pode escolher mais de uma opção como resposta)

( ) Limpeza frequente das mãos, utilizando álcool em gel ou água e sabão
( ) Rotina de limpeza e desinfecção de superfícies que foram expostas à pacientes suspeitos ou não
( ) Espaçamento entre os atendimentos agendados, evitando assim que os pacientes não se encontrem na sala de espera
( ) Orientação do uso de máscara em pacientes e acompanhante (quando indispensável)
( ) Uso de EPI por todos os integrantes da equipe
( ) Isolar paciente com a doença ou com suspeita em salas individuais adequadamente ventiladas
(...) Limpeza terminal diária

5. Considerando os casos que tem se apresentado como demanda espontânea ou urgência odontológica durante a pandemia de COVID-19, você:

( ) Considera atender clinicamente todos os casos porque se o paciente julgar necessário, sua vontade está em primeiro lugar
( ) Considera atender clinicamente todos os casos por motivos financeiros
( ) Considero atender os casos de urgência em compromisso ético ao bem estar do paciente
( ) Não considera atender qualquer caso porque o risco de contágio é muito grande

( ) A teleodontologia irá ser vista como uma melhor alternativa, se difundindo rapidamente e em maior escala

6. Com relação ao seu trabalho, devido a chegada da pandemia COVID-19, qual dessas opções MAIS SE APROXIMA da sua situação ATUAL?

( ) Permaneci atendendo e não houve mudança na minha rotina
( ) Adaptei meus serviços e não houve mudança na minha vida

( ) Não estou mais atendendo
( ) Estou em férias
( ) Estou trabalhando em home office
( ) Tive a jornada de trabalho e o salário reduzidos em 50%
( ) Tive a suspensão do contrato de trabalho com pagamento de seguro-desemprego
( ) Continuo trabalhando (nada foi alterado)
( ) Pedi demissão
( ) Fui demitido
( ) Outro

7. A empresa e/ou local onde você trabalha:
( ) Está aberta normalmente para o público
( ) Está aberta parcialmente para o público
( ) Está fechada para o público
( ) Está interditada/com acesso restrito de circulação de pessoas devido as medidas de distanciamento social/quarentena
( ) Não se aplica

8. Durante a pandemia as condições de trabalho e renda são:
( ) Melhores que a anterior
( ) Iguais a anterior
( ) Piores que a anterior

9. Em relação à sua renda:
( ) Não houve redução
( ) Houve redução de até 10%
( ) Houve redução acima de 10% até 50%
( ) Houve redução acima de 50% até 100%

10. As previsões econômicas apontam para um período de recessão. Diante disso, com base em suas reservas econômicas atuais, por quanto tempo você conseguirá se manter?
( ) Não tenho reserva econômica
( ) Menos de 1 mês
( ) 1 mês
( ) 2 meses
( ) 3 meses
( ) 4 meses
( ) 5 meses
( ) 6 meses
( ) Mais de 6 meses

DIMENSÃO 4 – ESTRESSE E ANSIEDADE

1. **Você está ou esteve em algum acompanhamento de saúde mental nos últimos 30 dias (Psicoterapia, atendimento com psicólogo ou psiquiatra)?**
   ( ) Sim
   ( ) Não

2. **Em tempos de pandemia do COVID-19, marque a alternativa que melhor descreve a frequência com que você sentiu os seguintes síntomas:**

   Por favor, leia cada afirmativa e indique a que que aconteceu a você na última semana. Não há respostas certas ou erradas. Não gaste muito tempo!

RESPOSTAS LIKERT segundo Vignola e Tucci, 2014.

( ) **Não aconteceu comigo durante a pandemia.**

( ) **Aconteceu comigo algumas vezes durante a pandemia? ou durante essa semana?**

( ) **Aconteceu comigo em boa parte do tempo desde o inicio da pandemia? ou durante essa semana?**

( ) **Aconteceu comigo na maior parte do tempo desde o inicio da pandemia? ou durante essa semana?**

- Dei-me conta que tinha a boca seca
- Senti dificuldade em respirar (ex: respiração excessivamente rápida ou falta de respiração na ausência de esforço físico)
- Senti tremores (por exemplo, das mãos ou das pernas)
- Preocupei-me com situações em que poderia vir a sentir pânico e fazer um papel ridículo
- Estive perto de entrar em pânico
- Senti o bater do meu coração mesmo quando não fazia esforço físico (ex: coração acelerado ou falhas no bater do coração)
- Tive medo sem uma boa razão para isso
- Tive dificuldade em me acalmar/descomprimir.
- Tive tendência para reagir exageradamente em certas situações
- Senti-me muito nervoso
- Senti que estava agitado
- Senti dificuldades em relaxar
- Fui intolerante quando qualquer coisa me impedia de realizar o que estava a fazer
- Senti que andava muito irritável

Table S3. Translated Questionnaire (English)

DIMENSION 1 – SOCIODEMOGRAPHIC CHARACTERISTICS OF THE INTERVIEWED POPULATION

Email: (Optional).

1. Which city do you live?

2. Which city do you work?

3. How do you identify yourself?
( ) Man
( ) Woman
( ) Other

4. According to the IBGE census categories for race or color, you declare yourself
( ) Indigenous
( ) Yellow
( ) Black
( ) Brown
( ) White
( ) Rather not answer

5. Age group
( ) 21 - 30 years old
( ) 31 - 40 years old
( ) 41 - 50 years old
( ) 51 - 60 years old
( ) Over 60 years old

6. Have you developed any signs and / or symptoms of COVID-19?
( ) I had symptoms
( ) I had no symptoms
( ) I don't know if I had symptoms

7. Were you diagnosed with the virus (SARS-CoV-2)?
( ) Yes, I got tested and the diagnosis was positive
( ) No, I got tested and the diagnosis was negative
( ) I had symptoms but I couldn't get tested

8. Has anyone in your family or work team developed any symptoms or been diagnosed with COVID-19?
( ) Yes
( ) Not
( ) I don’t know

DIMENSION 2- - EDUCATION CHARACTERISTICS OF THE INTERVIEWED POPULATION

1. Careers in Dentistry (You can choose more than one option as an answer)

( ) Operative Dentistry
( ) Dental Law and Ethics
( ) Pediatric Dentistry
( ) Orthodontics
( ) Oral Patology
( ) Oral Maxillofacial Prosthesis
( ) Dental prosthesis
( ) Family Health
( ) Dental Radiology and Imaging
( ) Temporo-Mandibular Dysfunction
( ) Occupational Dentistry
( ) Dentistry for Patients with Special Needs
( ) Functional Jaw Orthopedics
( ) Management
( ) Hospital Dentistry
( ) Teaching

2. Education

( ) General Dentistry
( ) Certificate
( ) Master's degre
( ) PhD
( ) Pos-doctoral training

3. For how long have you worked in clinical pratice?

( ) Up to 4 years
( ) 5 to 10 years
( ) 11 to 20 years
( ) 21 or more
( ) I am not in clinical practice

4. Where do you work? (You can choose more than one option as an answer)

( ) Public Hospital
(...) Private Hospital
(...) Basic Health Unit
(...) Private Dental Clinic
(...) Policlínica
(...) Research Laboratory
(...) Dental School
(...) Other

5. Do you know the dental practice and biosafety protocols related to COVID-19?

( ) Yes, all
( ) Some
( ) Only within my specialty
( ) Almost nothing
( ) None

6. Did you receive any training on the use, maintenance and disposal of PPE? If so, where was this training? More than one option can be checked. If you checked "no" in the question above, check "does not apply".

( ) Yes
( ) Not
( ) Graduate school
( ) Postgraduate studies
( ) In the institution / place where I work
( ) In another institution that I have worked
( ) On my own
( ) I saw a training video
( ) I follow the protocols of an Ebook
( ) Does not apply

7. Do you consult the dental council websites (CRO, CFO, among others) for information, clinical and biosafety protocols related to COVID - 19?

( ) Yes
( ) No

8. Where do you usually look for information on Biosafety and Health Waste Disposal?

( ) Books
( ) Journals
( ) Dental Council Protocols
( ) Internet
( ) Others

DIMENSION 3 - CLINICAL/WORK CHARACTERISTICS AND ECONOMICAL VARIABLES

1.Do you feel able to assist your patients with this information?

( ) Yes, totally
( ) Yes, but with doubts
( ) No

2. Which alternative describes how you feel about your job; more than one option can be marked as an answer:

( ) I need to go back to work to be able to pay my bills
( ) I need to go back to work because I love my profession and I can't stay without work, at home
( ) I will only go back to work if my boss demands or cuts my salary

( ) I am afraid of getting the disease by seeing my patients
( ) I am not afraid of catching the disease seeing my patients
( ) I didn't stop working during the Pandemic

3. In your dental practice routine, in relation to the use of PPE, mark what is true: DOTTED ANSWERS

I do not use
I used it and I will continue to use it
I started using after the pandemic
I have used it for lack of another option on the market

Surgical mask with triple TNT
Mask N95
Fabric mask
Disposable hat
Fabric hat
Protective goggles
Procedural gloves (disposable)
Fabric lab coat
Disposable lab coat
Acrylic face mask / FACE SHIELD
Disposable foot cover
Surgical Glove
Others___________________________

4.What have you done, or do you consider important to do in the dental practice environment, as a preventive measure in the transmission of coronavirus (You can choose more than one option as an answer)

( ) Frequent hand cleaning using hand sanitizer or soap and water
( ) Routine cleaning and disinfection of surfaces that have been exposed to patients
( ) Spacing between scheduled appointments, thus preventing patients from being in the waiting room
( ) Guidance on the use of a mask to patients and companions (when necessary)
( ) Use of PPE by all team members
( ) Isolate patient with the disease or with suspicion in individual rooms adequately ventilated
( ) Daily end of the day cleaning

5. Considering the cases that have been presented as spontaneous demand or dental urgency during the COVID-19 pandemic, you:

( ) Considers providing clinical care to all cases because if the patient deems it necessary, his will comes first
( ) Considers providing clinical care to all cases for financial reasons
( ) I consider providing clinical care to urgent cases in an ethical commitment to the patient's well-being
( ) Does not consider providing clinical care any case because the risk of contagion is very high

6. Regarding your work, due to the arrival of the COVID-19 pandemic, which of these options IS MOST APPROXIMATE to your CURRENT situation?

( ) I continued providing dental care and there was no change in my routine
( ) I adapted my services and there was no change in my life
( ) I'm not providing dental care anymore
( ) I'm on vacation
( ) I am working from home
( ) I had my workday and salary reduced by 50%
( ) I had the employment contract suspended with payment of unemployment insurance
( ) I keep working (nothing has changed)
( ) I quit
( ) I was fired
( ) Other

7. The company and / or place where you work:

( ) It is normally open to the public
( ) It is partially open to the public
( ) It is closed to the public
( ) It is forbidden / with restricted access for the circulation of people due to the measures of social distance / quarantine
( ) Not applicable

8. During the pandemic, working and income conditions are:

( ) Better than the previous one
( ) Same as previous
( ) Worse than the previous

9. Regarding your income:

( ) There was no reduction
( ) There was a reduction of up to 10%
( ) There was a reduction above 10% up to 50%
( ) There was a reduction above 50% up to 100%

10. Economic forecasts point to a period of recession. Given that, based on your current savings, how long will you be able to financily maintain yourself?

( ) I have no savings
( ) Less than 1 month
( ) 1 month
( ) 2 months
( ) 3 months
( ) Four months
( ) 5 months
( ) 6 months
( ) More than 6 months

DIMENSION 4 - STRESS AND ANXIETY

1. Are you or were you in any mental health follow-up in the last 30 days (psychotherapy, treatment with a psychologist or psychiatrist)?
( ) Yes
( ) No

In times of a COVID-19 pandemic, tick the alternative that best describes how often you felt the following symptoms: Please read each statement and indicate what happened to you in the last week. There are no right or wrong answers. Don't spend too much time!

LIKERT ANSWERS according to Vignola and Tucci, 2014.
( ) It didn't happen to me during the pandemic.
( ) Did it happen to me a few times during the pandemic? or during this week?
( ) Has it happened to me most of the time since the beginning of the pandemic? or during this week?
( ) Has it happened to me most of the time since the beginning of the pandemic? or during this week?

- I realized that my mouth was dry
- I had difficulty breathing (eg excessively rapid breathing or shortness of breath in the absence of physical exertion)
- I felt tremors (eg, of the hands or legs)
- I was worried about situations where I might feel panicked and make a fool of myself
- I came close to panicking
- I felt my heart beating even when I was not exerting myself (eg, racing heart or heart failure)
- I was afraid for no good reason.
- I had difficulty calming down/decompressing.
- I tended to overreact in certain situations
- I felt very nervous
- I felt I was agitated
- I found it difficult to relax
- I was intolerant when something prevented me from doing what I was doing
- I felt that I was very irritable
